# Late-in-life neurodegeneration after chronic sleep loss in young adult mice

**DOI:** 10.1093/sleep/zsab057

**Published:** 2021-03-26

**Authors:** Jessica E Owen, Yan Zhu, Polina Fenik, Guanxia Zhan, Patrick Bell, Cathy Liu, Sigrid Veasey

**Affiliations:** Department of Medicine, Perelman School of Medicine, University of Pennsylvania Philadelphia, Pennsylvania, USA

**Keywords:** sleep deprivation, partial sleep restriction, noradrenergic, pyramidal neurons, stereology, microglial dysfunction

## Abstract

Chronic short sleep (CSS) is prevalent in modern societies and has been proposed as a risk factor for Alzheimer’s disease (AD). In support, short-term sleep loss acutely increases levels of amyloid β (Aβ) and tau in wild type (WT) mice and humans, and sleep disturbances predict cognitive decline in older adults. We have shown that CSS induces injury to and loss of locus coeruleus neurons (LCn), neurons with heightened susceptibility in AD. Yet whether CSS during young adulthood drives lasting Aβ and/or tau changes and/or neural injury later in life in the absence of genetic risk for AD has not been established. Here, we examined the impact of CSS exposure in young adult WT mice on late-in-life Aβ and tau changes and neural responses in two AD-vulnerable neuronal groups, LCn and hippocampal CA1 neurons. Twelve months following CSS exposure, CSS-exposed mice evidenced reductions in CA1 neuron counts and volume, spatial memory deficits, CA1 glial activation, and loss of LCn. Aβ _42_ and hyperphosphorylated tau were increased in the CA1; however, amyloid plaques and tau tangles were not observed. Collectively the findings demonstrate that CSS exposure in the young adult mouse imparts late-in-life neurodegeneration and persistent derangements in amyloid and tau homeostasis. These findings occur in the absence of a genetic predisposition to neurodegeneration and demonstrate for the first time that CSS can induce lasting, significant neural injury consistent with some, but not all, features of late-onset AD.

**Statement of Significance**

Chronic curtailment of sleep is common in modern society, yet the long-term consequences on brain health are unknown. Our results demonstrate that chronic short sleep in the young adult mouse imparts irreversible degenerative changes in at least two brain regions susceptible to Alzheimer’s disease, the locus coeruleus and hippocampus, present a year after sleep loss exposure. Moreover, a genetic predisposition is not necessary for chronic short sleep to induce significant lasting injury.

## Introduction

There are approximately 50 million individuals worldwide with dementia, most of whom have Alzheimer’s disease (AD) [1], and as the population ages, the number of individuals with AD is expected to increase significantly [2]. Unfortunately, interventions to effectively treat or prevent AD remain elusive [3]. Several clinical studies indirectly support the possibility that insufficient sleep may negatively influence AD. Specifically, poor sleep quality and short sleep duration are associated with amyloid-β (Aβ) plaque burden and cognitive impairment [4–6], while sleep disruption predicts a more rapid cognitive decline and incident dementia [7]. Chronic short sleep (CSS), defined as ≤6 h sleep/night, is present in approximately 10% of American adults and is also observed commonly in many other developed countries [8, 9]. Critically, the number of working adults regularly experiencing CSS has increased over the last few decades [10]. Thus, determining whether CSS can impart lasting significant neural injury or hasten the development of any features of AD is of heightened importance.

AD is defined pathologically by the presence of extracellular amyloid plaques, intraneuronal aggregates of hyperphosphorylated tau protein and significant brain atrophy. Although specific mechanisms by which the pathologies occur are not known, a largely accepted hypothesis is the amyloid cascade hypothesis, which posits that increases in Aβ set off a chain of events including glial inflammation, tau modifications and degeneration [11–13]. A substantive body of research implicates soluble Aβ and tau oligomers in AD neural injury, more so than the diagnostic pathological aggregates [14–19]. There is also evidence that soluble Aβ and tau may act additively and/or synergistically in synaptic dysfunction, cognitive impairments and neuron loss [19–21]. Intriguingly, sleep deprivation in both humans and mice has been shown to acutely increase levels of Aβ and tau in the hippocampal extracellular space in mice and cerebrospinal fluid in humans [22–24]. Wake elevations in Aβ and tau both appear to occur in part due to increased neuronal activation in wakefulness [24–26]; although slowing of clearance across the interstitial space in wakefulness may also contribute [27]. Recently an Aβ feed-forward cycle has been described in the hippocampus (an AD-vulnerable brain region), where increased Aβ increases neuronal activation, which in turn increases Aβ production [28, 29]. Notably, earlier studies in WT rats showed that total sleep deprivation across several weeks resulted in systemic injury and ultimately death, yet minimal effects were evident in the brain immediately following sleep deprivation [30]. We, therefore, hypothesized that CSS exposure in the young adult mouse might initiate a slow, but progressive, Aβ and tau dyshomeostasis, manifesting as insidious neural injury in AD-susceptible brain regions late-in-life.

## Methods

### CSS protocols

**Figure 1.**
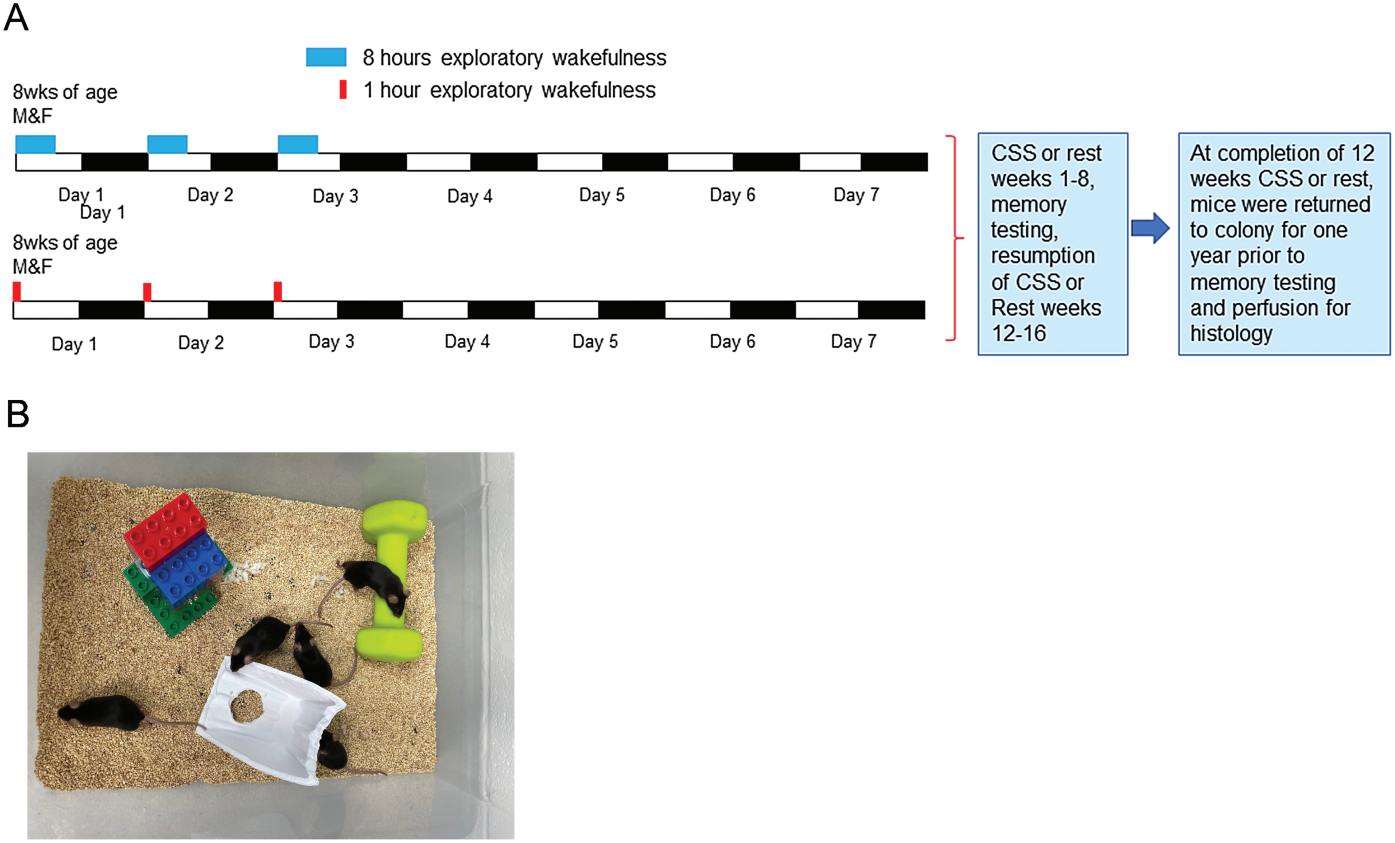
Study design overview and experimental setting. (A) The Chronic short sleep (CSS) experimental paradigm week for sleep loss (upper) and rested control (lower). All mice are maintained throughout the study on the same 12 h:12 h light−dark schedule (white, black bars). Mice were randomized to 8 h of sleep loss (CSS, light blue bars) or 1 h in the same environment (rested controls, red bars) at the beginning of the lights-on period for 3 consecutive days each week. At the completion of exposures and on non-exposure days mice were returned to the animal colony. This pattern was performed for 8 consecutive weeks. Mice were then given a 4-week long break during which spatial memory testing was performed, followed by 4 consecutive weeks of CSS or rested conditions. All mice were returned to the animal colonies for 12 months prior to repeated memory testing and perfusions for histology. (B) Overhead image of one of the enriched environments for the study with three climbable objects. Additional object were substituted in when any mouse was observed to be still.

Studies were performed at the University of Pennsylvania in accordance with the National Institutes of Health Office of Laboratory Animal Welfare Policy and the University of Pennsylvania’s Institutional Animal Care and Use Committee. Male and female C57BL/6J (Jackson Laboratory) mice were studied with equal numbers of each sex used in all experiments. Mice were 8−9 weeks at the start of CSS or rest control conditions. Figure 1, A summarizes the complete study protocol. CSS was performed by placing mice and their littermates in novel enriched environments (as shown in Figure 1, B), where climbing toys were exchanged whenever a mouse in the paradigm became quiescent [31, 32]. Across the CSS exposures, mice were continuously visualized by one or two researchers, each watching mice directly for 1–2 h shifts to ensure eight continuous hours of active wakefulness in each CSS mouse. This CSS paradigm does not elevate blood corticosterone levels [33]. CSS exposures (five females and five males) occurred on 3 consecutive days of each week for the first 8 h of the lights-on period, when mice typically sleep. Using this same paradigm, we have previously reported the long-term effects on sleep after CSS to include a small increase in wake time during the lights-on period and a blunted diurnal ratio for wake activity [34]. Rested controls (five females and five males) were exposed to the same environment for 1 h/day for 3 days/week for 12 weeks. CSS and rested control conditions were administered for 12 weeks within a 16 week period (weeks 1–8 and weeks 12–16) with mice returned to their home cages after each sleep loss exposure and at the end of the 12th week of CSS or rest conditions for 12 uninterrupted months to be examined at 18 months of age (CSS_12wk_ + Rec_12mo_). Throughout the study, including CSS exposures, mice were maintained on the same 12 h:12 h light:dark schedule, same ambient lighting and temperature, and fed ad libitum standard rodent chow and water.

### Histology, microscopy and stereology

At 18 months of age, 12 months after the last CSS exposure, mice were anesthetized with sodium pentobarbital and transcardially perfused with 4% paraformaldehyde. Extracted brains were cryopreserved, coronally sectioned at 60 µm, and collected in sequential 1:6 series in 24-well plates [35]. For immunohistology, selected sections were blocked in Triton−PBS−1% BSA ± mouse IgG, followed by incubation with primary antibodies diluted in blocking buffer for 1–3 days at 4°C. Primary antibodies used were: *Aβ42*, AB5078P Millipore; *Aβ42* (12F4), 805501 BioLegend; *AT8* tau, (P-Ser202/Thr205), MN1040 Thermo Fisher; *CD68*, Ab125212 Abcam; *GFAP*, 13–0300 Thermo Fisher; *Iba-1,* Ab107159 Abcam; Norepinephrine transporter (*NET*), 1447-NET Phosphosolutions; *P231tau*, phospho-T231 tau, Ab151559 Abcam; and Tyrosine hydroxylase (*TH*) LS-C124752 LSB. Both Aβ _42_ antibodies target the carboxy-terminus of Aβ _42_, and thus do not detect full-length amyloid precursor protein. Sections from APP− ^/^− (B6.129S7 *APP*^*tm1dbo*^/J and Tau− ^/^− (B6.129X1 *mapt*^*tm1Hnd*^/J) mice were used (both from Jackson Laboratory) to confirm Aβ and tau antibody specificity. A mouse strain with robust amyloid plaques (B6SJL-Tg(APPSwFlLon,PSEN1*M146L*L286V)6799Vas/Mmjax, Jackson Laboratory) was used as a positive control for Thioflavin labeling as below. No primary antibody controls were used to normalize for non-specific labeling and autofluorescence. For light microscopy immunohistology, secondary antibodies were labeled with Vector blue alkaline phosphatase. For confocal microscopy, secondary antibodies for immunofluorescence were conjugated with Alexa Fluor probes: 488, 555, 594 and 647(Invitrogen). Imaging was performed with Leica DM5500B (light microscopy) and DM4B (stereology) and Leica SP5/AOBS (confocal). Confocal laser intensities, nm range, detector gain, exposure time, amplifier offset, and depth of the focal plane within sections per antigen target were standardized across compared sections [35]. Thioflavin-T (T3516, Sigma-Aldrich) was used according to the manufacturer’s protocol to detect amyloid plaques and fibrils in two dry-mounted sections/mouse of hippocampus and rostral cortex (Bregma AP −2.30 mm and 1.34 mm, respectively). Image analysis was performed using ImageJ software on converted 8-bit grayscale inverted images, with a detection threshold standardized across all images to detect percent areas and synaptic protein densitometry.

Microglial dystrophy in the hippocampus has been defined in AD by the presence of fragmented, de-ramified, beaded and/or spheroidal microglial processes [36]. These characteristics were used to define the percentage of microglial cells in CA1 and CA3 with any dystrophic features within the bilateral CA1 and CA3 regions of two mid-HC section/mouse (Bregma AP −1.8–2.0 mm). Counts were obtained from confocal microscopy images acquired across 4 µm, using 40× oil magnification to obtain six images per mouse.

Total LCn count estimation of TH antibody labeled neurons with Giemsa stained nuclei was performed using optical fractionator stereology [37] modified for the LC using a 1:2 series of sections (three of six LC wells, covering Bregma AP −5.02 mm to −5.80 mm) twelve months following CSS or Rest exposures (*n* = 4−7 mice/group), using our previously described protocol [33]. Stereology was performed using a Leica DM4B microscope equipped with a Stereo Investigator workstation (MicroBrightField, v.11.09) [37]. The 100× oil objective was used to count cells, within the probe boundaries, with nuclear chromatin in TH-labeled cytoplasmic cell bodies with diameters >15 µm. A sampling scheme with a 0.25 area sampling fraction and 0.80 thickness sampling fraction was used. This strategy provided >200 counts/mouse across 8–9 sections and Gundersen coefficients of error <0.10 for every animal. Scorers were blinded to age and sleep conditions.

CA1 neuron count estimates were performed with optical fractionator stereology counts of Cresyl violet labeled nucleoli in pyramidal neurons (with no additional staining) [37], using a 1:3 series of sections (using two wells of hippocampal level sections, covering Bregma AP −0.82 to −4.04 mm), incorporating an area sampling fraction of 0.004 (9.5 × 9.5 µm counting frame = 90.25 µm^2^*a*_frame_, 150 × 150 µm distance between counting frames = 22,500 µm^2^*a*_step_, *a*_frame_ /*a*_step_ = 0.004 area sampling fraction) and a thickness sampling fraction of 0.79 (dissector height 15 µm × average mounted section thickness 19.1 µm). This sampling strategy in CA1 also provided in >200 counts/mouse and Gundersen coefficients of error <0.10.

Hippocampal (HC) (CA1, CA2, CA3, and dentate gyrus, DG) and lateral ventricular volumes were measured in CSS_12wk_ + Rec_12mo_ mice and rested controls using the Cresyl violet stained sections used for stereology. We implemented a Cavalieri estimator approach using the same 1:3 HC series of coronal 60 µm sections through the hippocampus (Bregma −0.82 to −4.04). Using the Stereo Investigator workstation, regions of interest were outlined as previously described [38]; areas were measured, volumized for section original thickness, and corrected for the 1:3 sampling. Lateral ventricle volumes were measured across the same set of sections selected for HC volumes.

### Object-place memory recognition test

Rest and CSS mice were assessed at 4 months of age (after 8 weeks CSS and 3 days recovery) and again at 18 months (after 12 weeks CSS and 12 months recovery) for a hippocampal-dependent learning/memory task (spatial object recognition). The spatial recognition protocol was adapted from a detailed published protocol [39]. We performed the test in red light at the beginning of the lights-on period. Mice were handled and habituated to handlers and the experimental environment (60 cm × 50 cm × 30 cm) without objects, 10 min for three consecutive mornings. One day prior to testing tails were marked to distinguish each mouse within a single cage. On the test day, mice were video recorded as they received a 10 min training session, followed by a test session 90 min later in which one of the two objects was moved. Between training and testing sessions mice were left unperturbed in their home cages. Scorers reviewing videos were blinded to the conditions of mice. Place preference for each of the two objects was determined as the percentage time (3 min) spent in the observation field attending to the object to be moved, relative to both objects (trial), and percentage of time with the moved object, relative to time spent with both objects (test).

### Statistical analysis

All statistical analyses were performed using GraphPad statistical software (Prism, version 6.0). To compare distinct CSS and rested responses in WT mice, for normally-distributed data (Shapiro–Wilk normality test confirmed) unpaired *t*-tests were used. For non-normal data sets, the Mann–Whitney *U* test was performed. When multiple brain regions were analyzed, one-way ANOVA was used, implementing Sidak’s post hoc analysis to control for multiple comparisons. Within-animal Pearson’s linear correlations were performed for CA1 versus LCn counts within animals and CA1 cell counts and CA1 volumes. Within animal memory testing was performed using repeated-measures two-way ANOVA with Sidak’s post-hoc (*t*) analyses. To analyze the effects of brain region (CA1, CA3) and sleep condition (rest, CSS), two-way ANOVA was used with Tukey’s pre-specified multi-comparison post hoc (*q*) test. The cutoff for significant statistical power for all analyses was a corrected *p* < 0.05.

## Results

### HC and LCn degeneration are evident 1 year after early adulthood CSS

**Figure 2.**
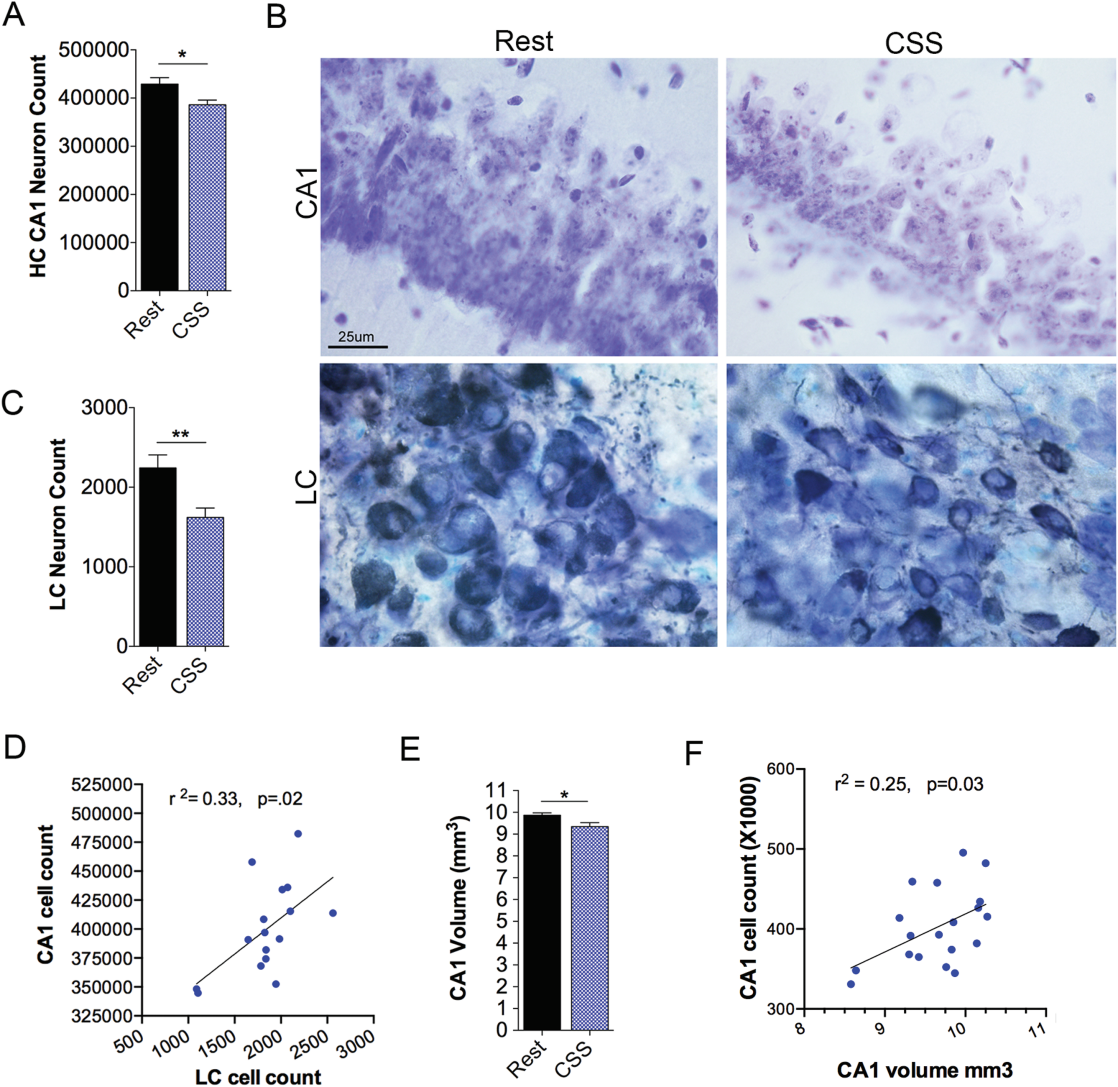
CA1 hippocampus (HC) and locus coeruleus (LC) degeneration is evident 1 year after CSS exposure. CA1 neurons and volume and LCn were examined 12 months after CSS or Rest exposures. (A) Optical fractionator stereological counts of CA1 pyramidal neurons in controls (Rest, black bars) and CSS exposed mice (CSS, blue bars), *t* test, *n* = 9–10 mice/group. (B) Representative images of Cresyl violet stained sections in CA1 (upper panels) and tyrosine hydroxylase (dark blue) labeled LC neurons counterstained with Giemsa in Rest and CSS mice (lower panels) 1 year after CSS and Rest exposures. (C) Optical fractionator stereological counts of LCn in the same groups as in A, Mann−Whitney *U*, *n* = 4–7 mice/group. (D) Within-animal relationship between CA1 neuron number and LCn number with linear regression best fit line (*n* = 16 mice). (E) Cavalieri’s estimator volumes of CA1 (*t* test, *n* = 9–10/group) in control (Rest, black bars) and CSS-exposed (CSS, blue bars) mice also 12 months following CSS or Rest exposure. (F) Within-animal Pearson’s relationship (*n* = 19 mice) of CA1 cell count and CA1 volume with linear regression best fit line. Data in A, C, and E are presented as mean ± *SE*, *p < 0.05 and **p < 0.01. Calibration marker, B, 25 μm for all panels.

The CA1 subregion of the HC is particularly vulnerable to early injury and degeneration in AD [40]. Loss of HC neurons in AD can be accurately assessed using stereological estimates of non-immunolabeled stained neurons [41]. We, therefore, performed CA1 neuron counts using optical fractionator stereology to count Cresyl violet-labeled pyramidal nuclei in mice at age 18 months, 12 months after CSS. Experiments were not sufficiently powered (7%) to discern sex-dependent differences with *n* = 4–5 mice/sex and sleep condition). Thus, responses are reported as collective males and females. Remarkably, young adulthood CSS exposure resulted in a reduction in CA1 neurons at 18 months (*df* = 19, *t* = 2.6, *p* < 0.018, Figure 2, A). Representative examples of CA1 pyramidal neurons in Rested and CSS-exposed mice are shown in Figure 2, B. Noradrenergic locus coeruleus neurons (LCn) are also susceptible to early injury and degeneration even in the prodromal phase of AD [42], and previously we found that CSS results in loss of LCn, detected 4 weeks after CSS [34]. Several mice had LC sections unsuitable for stereological counts, due to cracks in the tissue near the fourth ventricle. LCn counts were reduced 12 months after CSS (*df* = 14, *U* = 4.0, *p* = 0.002, Figure 2, B and C). Notably, within animal LCn counts predicted CA1 counts (linear regression, *df* = 14, *r*^2^ = .33, *p* < 0.019, Figure 2, D). To lend further support for degeneration within the CA1, we next examined CA1 volume, finding a reduction in CA1 volume (*df* = 19, *t* = 2.4, *p* < 0.05, Figure 2, E). The within animal CA1 cell counts predicted within animal CA1 volume (*df* = 17, *r*^2^ = 0.25, *p* < 0.030, Figure 2, F).

**Figure 3.**
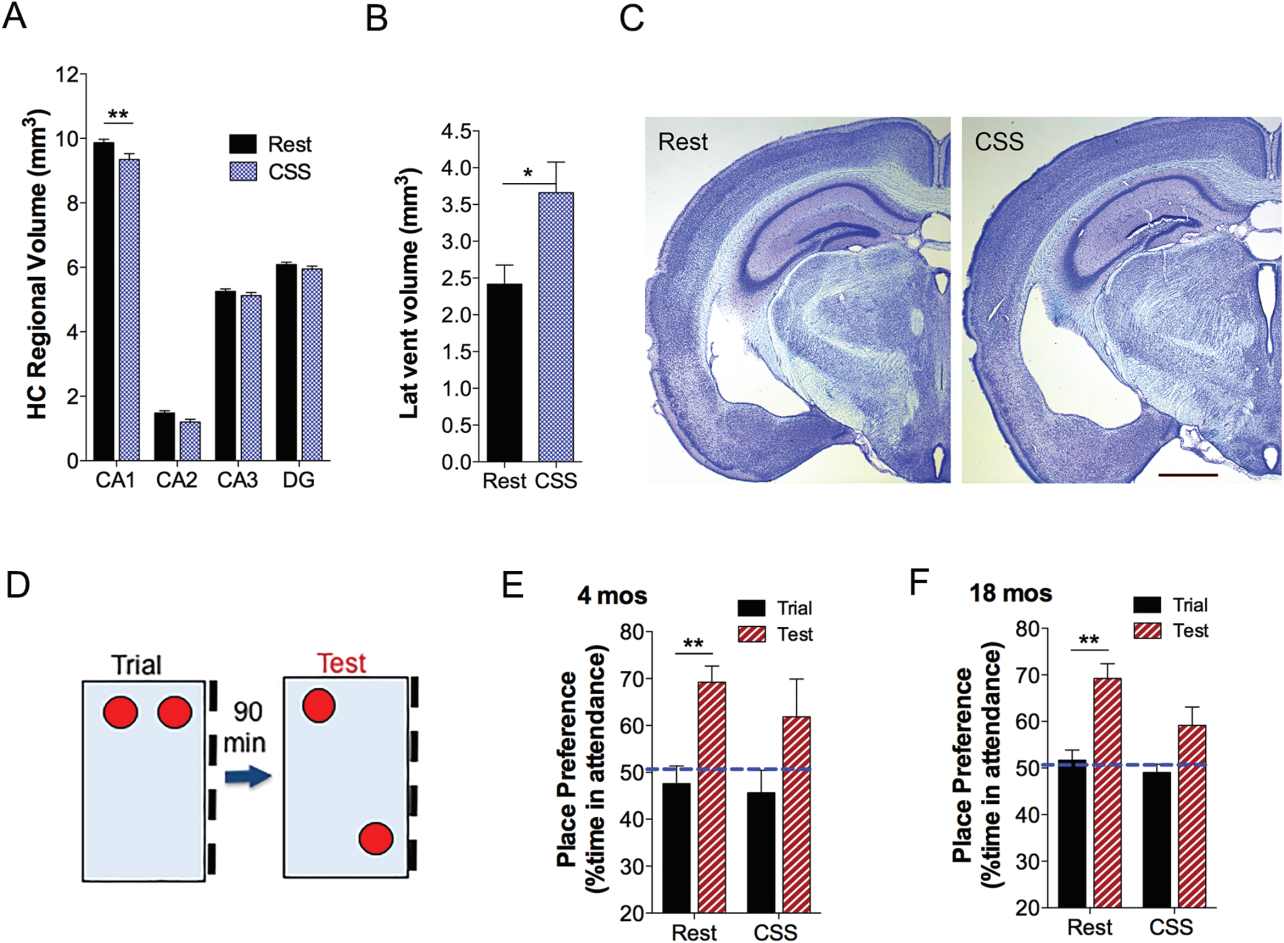
Volumetric and memory effects of CSS. (A) Cavalieri’s estimator volumes for HC subregions CA1, CA2, CA3, and dentate gyrus (DG) (*n* = 9–10 mice/group) were compared in controls (Rest, black bars) and CSS-exposed mice (CSS, blue bars) at 18 months of age (12 months after CSS, Rest conditions), one-way ANOVA, with overall CSS effect (*F* = 891, *p* < 0.0001). (B) Cavalieri volumes for lateral ventricles (Lat Vent, C) in the same mice HC subregions were measured, unpaired *t*-test. (C) Cresyl violet stained 60 μm coronal sections in Rest (left) and CSS-exposed mice (right) at Bregma AP −2.18 mm. (D) Diagram illustrating the training/testing environment for spatial object recognition testing. Training (Trial) and testing conditions (Test) were interspersed with a 90 min interval in home cage. (E, F) Percentage of time spent attending to the object prior to moving (trial, black bar) and after moving (test, red/white bar) in mice exposed to Rest or CSS conditions at 4 months of age (after 8 weeks CSS, E, *n* = 7–10 mice/group) and 18 months of age (after 12 weeks CSS and 1 year recovery, F, *n* = 9–10 mice/group), measured with two-way ANOVA. Data are presented as mean ± *SE*. *p < 0.05; **p < 0.01. Calibration bar, C, 1 mm.

We next turned to address whether CSS injury occurs similarly throughout sub-regions of the hippocampus, or whether only select regions evidence injury, as is evident in AD. Here, we compared rest and CSS volumes in CA1, CA2, CA3, and DG, using ordinary one-way ANOVA with the Sidak post-hoc *t*-test. Overall, there was an effect of CSS on volumes (*F* = 891, *p* < 0.0001); however, the only region showing a reduction in volume was CA1 (*df* = 85, *t* = 3.5, *p* < 0.003, Figure 3, A). While examining HC sub-region volumes and CA1 cell counts we noted incidentally larger lateral ventricles in some of the CSS mice. Using Cavalieri’s estimator approach, we measured lateral ventricle volumes in CSS and Rest mice. CSS mice, relative to age-matched Rest control mice exhibited large lateral ventricles (*df* = 19, *t* = 2.5, *p* = 0.022, Figure 3, B). Examples of the ventricular area at Bregma AP −2.18 mm are presented in Figure 3, C.

### Spatial learning/memory impairments persist 1 year after CSS

Spatial contextual memory is dependent on HC function [43, 44], and notably smaller HC volumes predict memory impairment in both mild cognitive impairment and AD [45]. Using a spatial object recognition assay, as illustrated in Figure 3, D, mice were tested at 4 months of age (after 8 weeks CSS or Rest exposures) and again at 18 months of age following CSS_12wks_ + Rec_12mos_ or Rest conditions. At 4 months of age, there was an overall trail-test factor (*df* = 15, *F* = 16.5, *p* = 0.001). The 4-month-old rested mice showed place preference for the moved object (*df* = 15, *t* = 3.6, *p* = 0.005), while CSS exposed mice did not (*df* = 15, *t* = 2.3, *p* = 0.076, Figure 3, E). Similarly, there was an overall time factor for mice at 18 months of age (*df* = 17, *F* = 17.8, *p* = 0.001). At 18 months of age rested mice showed place preference for the moved object (*df* = 17, *t* = 3.7, *p* < 0.004), while CSS-exposed mice showed no preference for the moved object (*df* = 17, *t* = 2.2, *p* = 0.076, Figure 3, F). Thus, spatial learning and/or memory impairments develop following CSS and persist late-in-life.

### Aβ _42_ is increased in CA1 1 year after CSS, co-localizing with microglia, but not evident as plaque

**Figure 4.**
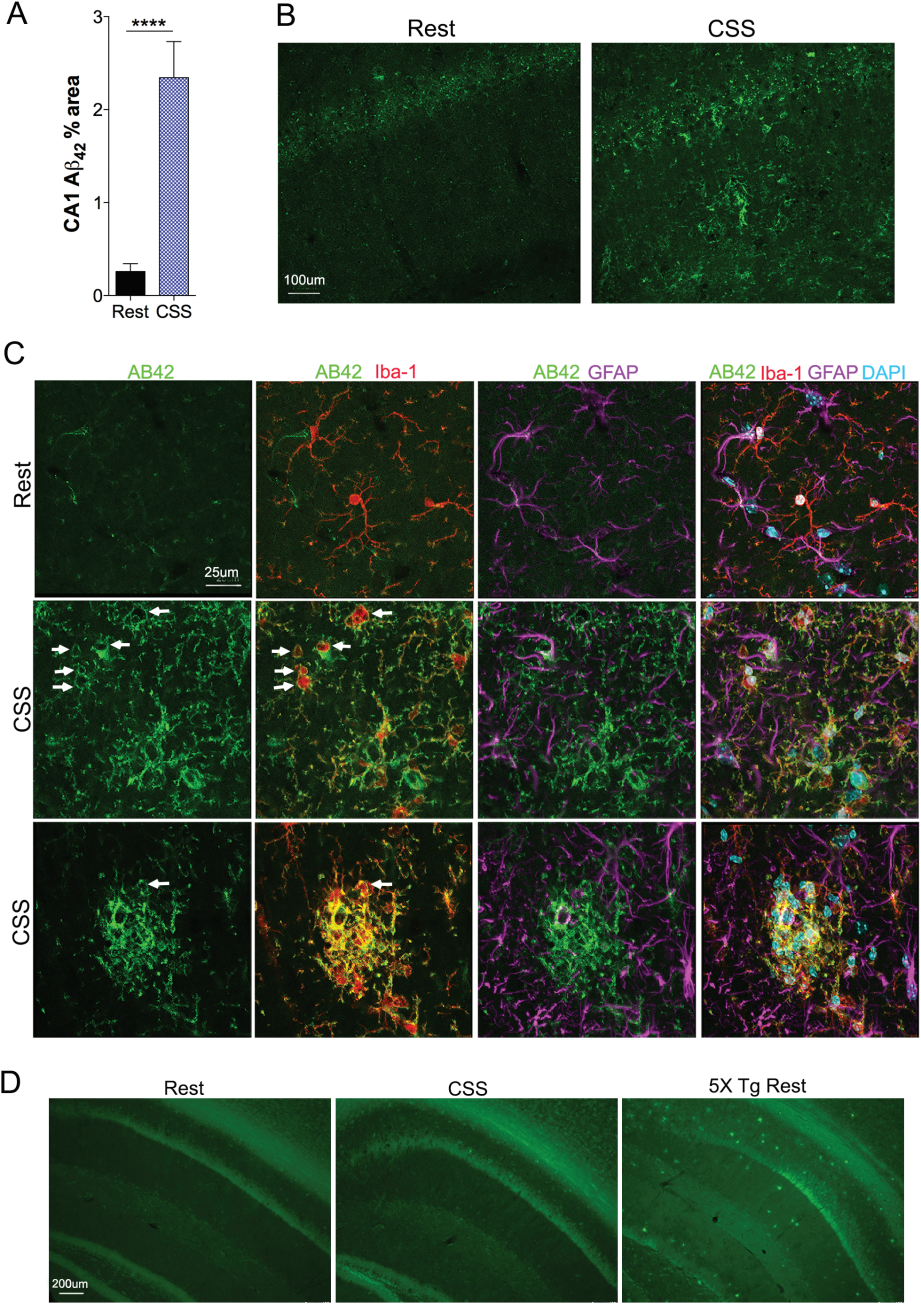
Aβ _42_ is increased in CA1 1 year following CSS exposure and localizes with microglia. (A) Aβ _42_ immunoreactivity in CA1in mice at 18 months of age, 12 months after exposure to Rest (black bar and CSS conditions (blue bar) for *n* = 9–10/group, analyzed with an unpaired *t*-test. (B) Representative confocal images of Aβ _42_ (488, green) punctate patterns in CA1. (C) High power confocal images in CA1 to show relationship between Aβ _42_ (green) and microglia (Iba-1, 594, red) and astrocytes (GFAP, 647 purple) with upper row (rest) and lower two rows (CSS). Arrows highlight Aβ _42_ outlining membranes of Iba-1 microglia. (D) Thioflavin-T fluorescence (488, green) in HC in mice at 18 months after Rest (left panel), CSS (middle panel) and positive control AD murine model 5XTg (right panel). Data are not summarized in graph form, as no thioflavin was detected in any Rest or CSS WT mice. Calibration bars, B, 100 μm; C, 25 μm; and D, 200 μm.

Prior to the development of amyloid plaque in mouse models of AD, a punctate pattern of Aβ _42_ may be observed in several brain regions including the HC in AD transgenic mice [46], suggesting that this punctate pattern of Aβ _42_ in CA1 is a pre-plaque finding in AD. Here, we found that young adult CSS exposure resulted in a marked increase in late-in-life CA1 punctate Aβ42 immunoreactivity (*df* = 18, *U* = 8.0, *p* < 0.0001, Figure 4, A. Representative confocal images of Aβ _42_ immunoreactivity 12 months after rest and CSS conditions are presented in Figure 4, B. We next determined whether Aβ _42_ co-localized with glial cells. A clear relationship was evident in all mice, where Aβ _42_ co-localized with Iba-1+ microglial cells (Figure 4, C). In contrast, Aβ _42_ did not show increased co-localization with GFAP. Two of the CSS mice showed clusters of microglial cells also with strong Aβ _42_ co-localization, suggestive of diffuse amyloid plaques. In contrast, astrocytes were absent from the microglia and Aβ _42_ clusters and tended to surround areas of intense Iba-1 and Aβ _42_ labeling. To ascertain whether the increased Aβ after CSS formed fibrillization, sections were labeled with Thioflavin-T. Thioflavin-T labeling of fibrils was not detected in either Rest of CSS exposed mice in the HC or rostrally, while robust signaling was evident in the 5×Tg AD mouse model (positive control); images are provided in Figure 4, D.

### Phosphorylated tau is upregulated in the CA1 and entorhinal cortex 1 year after CSS.

**Figure 5.**
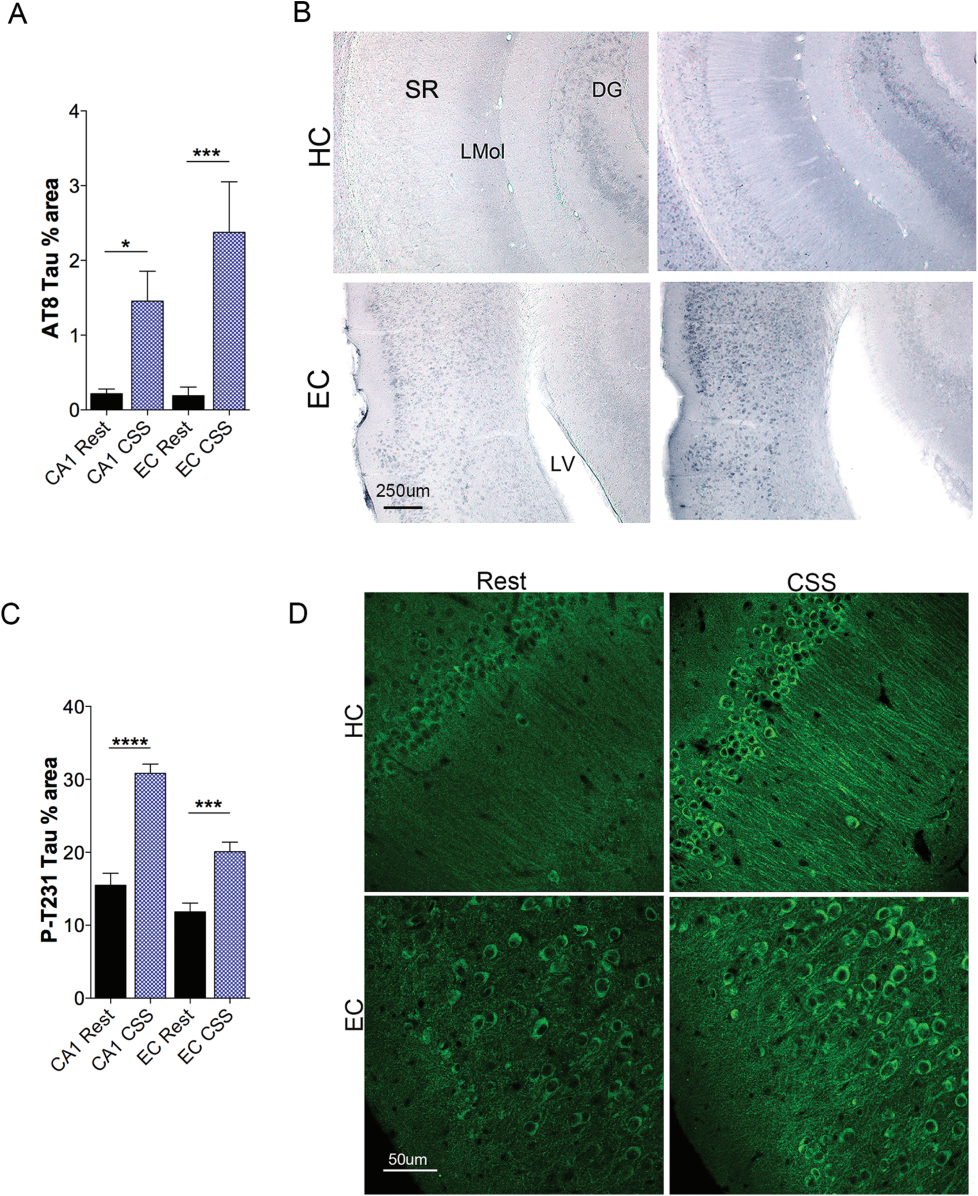
Increased tau phosphorylation is evident in CA1 and entorhinal cortex (EC) 1 year following CSS exposure. (A) Area percentage of AT8 tau (PSer202/Thr205) immunoreactivity in CA1 and EC (*n* = 9–10/group) in Rest (black bar) and CSS (blue bar). (B) Representative image of AT8 immunoreactivity (blue) in HC (upper panels) and EC (lower panels) in Rest and CSS mice. (C) Area percentage of tau with phosphorylation at Thr231 (P-T231) tau immunoreactivity in CA1 and entorhinal cortex (EC) in Rest (black bars, *n* = 9) and CSS exposed mice ((blue bars, *n* = 10). (D) Representative confocal images of P-T231 tau (488, green) in HC (upper) and EC (lower) in Rest and CSS-exposed mice 12 months after CSS and Rest exposures. Data (A, C) were analyzed with one-way ANOVA and where overall CSS effects were observed Sidak’s post-hoc tests were performed. (D) *p < 0.05; ***p < 0.001; ****p < 0.0001. SR, stratum radiatum; LMol, laconosum moleculare; DG, dentate gyrus; LV, lateral ventricle. Calibration bars, B, 250 µm and D, 50 μm.

In transgenic (P301S) tauopathy mice, CSS results in sustained phosphorylation tau (p-tau) in the HC, particularly in CA1 [47]. The entorhinal cortex (EC) is considered an early site of tau phosphorylation in tauopathies, including AD [48] and is considered the source of HC p-tau [49]. AT8 antibody recognizes tau isotopes P-Ser202 and P-Thr205 and is used for Braak staging in AD [50]. Thus, we measured AT8 immunoreactivity in both the CA1 and EC. An overall ANOVA CSS-interaction was identified for AT8 immunoreactivity, degrees of freedom divisor (DFd) = 35, *F* = 8, *p* = 0003. AT8 immunoreactivity in the HC was increased in CSS-exposed mice (*df* = 35, *t* = 2.4, *p* = 0.043, Figure 5, A), and EC AT8 increased in mice exposed to CSS (*df* = 35, *t* = 4.2, *p* = 0004, Figure 5, A). Representative AT8 images for both CA1 and EC are shown in Figure 5, B. A second phosphorylation modification of tau, P-Thr231, is observed in higher levels in the cerebrospinal fluid of persons with AD, relative to cognitively normal age-matched adults [51]. This post-translational modification imparts a *cis* form of tau that is not only neurotoxic but may spread to other brain regions [52]. Thus, we examined whether CSS also influenced levels of P-Thr231 in both the EC and CA1 HC. Overall CSS differences were observed for P-Thr231 immunoreactivity, *F* = 35, *p* < 0.0001. P-Thr231 tau was increased in response to CSS, relative to rest controls in both the CA1 (*df* = 35, *t* = 7.9, *p* < 0.0001) and EC (*df* = 35, *t* = 4.2, *p* = 0.0001), summarized in Figure 5, C. A representative image for each region and condition is shown in Figure 5, D. In summary, 1 year after exposure to CSS across young adulthood in WT mice, tau hyperphosphorylation is evident in regions of the brain vulnerable to p-tau changes in AD.

### CA1 glial activation and is evident 1 year after CSS exposure

**Figure 6.**
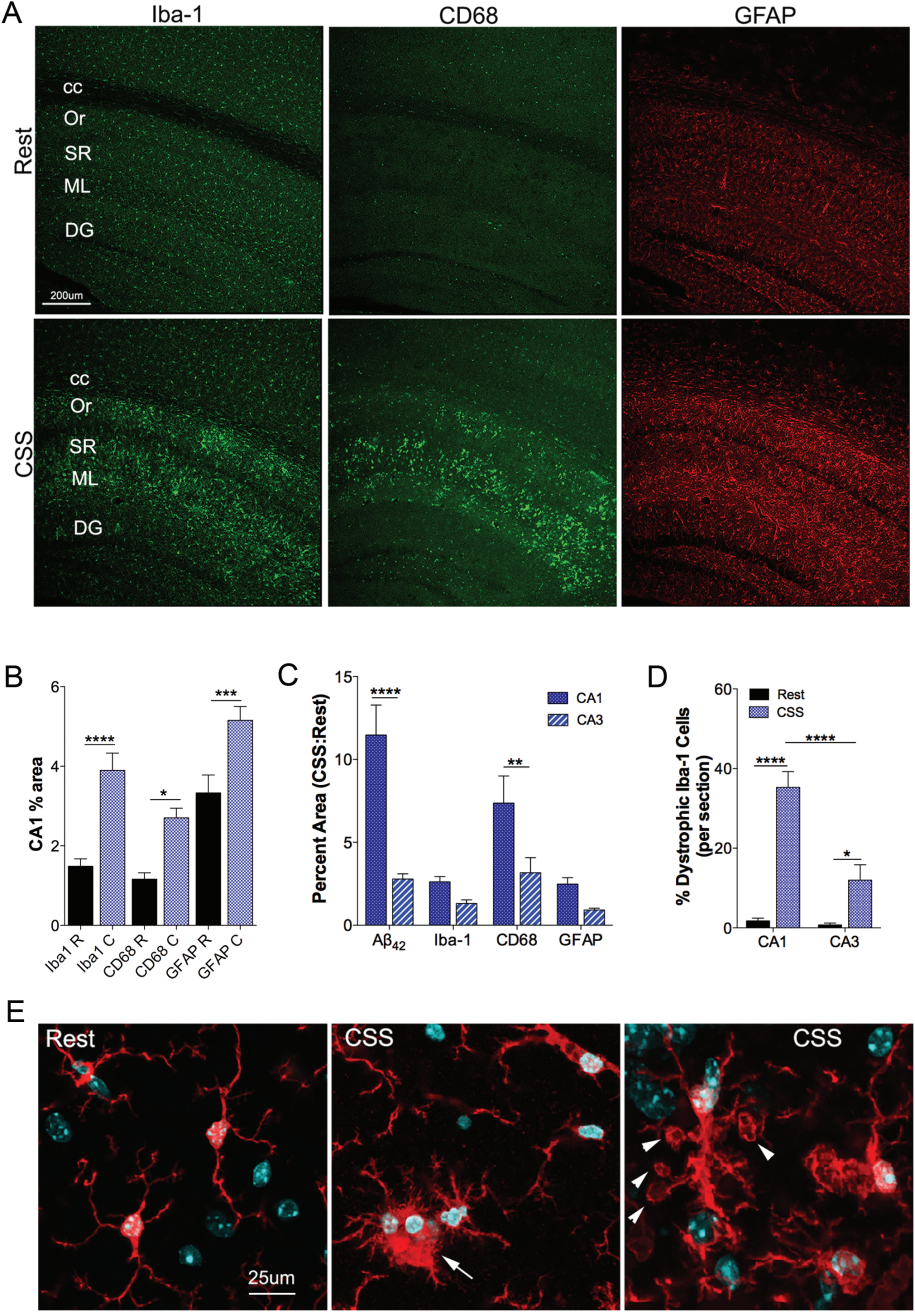
Late-in-life glial activation is evident in the HC after young adult CSS. (A) Confocal images of HC at Bregma AP −2.18 mm showing Iba-1 (488, green, left panels), CD68 (488, green, middle panels), and GFAP (594, red, right panels) labeling in mice 1 year after CSS (lower panels) and Rest (upper panels) exposures. (B) Percentage area in CA1 for Iba-1, CD68 and GFAP across Rest (black bars) and CSS conditions (blue bars). (C) Percent increase (relative to Rest) in CSS-exposed mice in the area covered for Aβ and glial markers (Iba-1, CD68 and GFAP), comparing CA1 (dark blue) and CA3 (blue/white striped). (D) Percentage of Iba-1-labeled microglia with dystrophic features in CA1 and CA3 in CSS-exposed mice. (E) High power confocal images of Iba-1-labeled (red) microglia with DAPI (blue) labeled nuclei in Rest (left panel) and CSS (middle and right panels) mice reveal dramatic changes in morphology of microglia. Arrow highlights an amoeboid microglial cell (middle panel) and arrow heads reveal vacuolar changes in CSS microglia (right panel). CC, corpus callosum; Or, oriens layer, SR, stratum radiatum; ML, moleculare lacanosum, DG, dentate gyrus. Data are mean ± *SE* and were analyzed in B, D by one-way ANOVA using Sidak’s post hoc comparisons for sleep condition effects and in C by two-way ANOVA with selected post-hoc Tukey analyses for region. *p < 0.05, ***p < 0.001; ****p < 0.0001. Calibration bars, A, 200 µm, C, 25 µm.

Glial responses in CA1 were also examined 12 months after CSS exposures to relate to cell and volume loss and Aβ and p-tau changes. Both Iba-1 and CD68 were examined to characterize the microglial response to CSS in CA1, and GFAP was examined for astrocyte response. Using one-way ANOVA to test CSS effects on the three glial markers, we observed overall differences across sleep conditions, DFd = 51, *F* = 11, *p* < 0.0001. A robust increase in Iba-1 percentage area was observed in response to CSS (DFd = 51, *t* = 5.2, *p* < 0.0001 Figure 6, A and B). CD68 also increased in response to CSS (DFd = 51, *t* = 2.9, *p* = 0.0124, Figure 6, A and B). To characterize the astrocyte response to CSS, GFAP percentage area was measured in CA1 across rest and CSS mice. CSS resulted in increased GFAP labeling in CA1 (DFd = 51, *t* = 3.9, *p* = 0.0004, Figure 6, A and B). These striking increases in Iba-1, CD68, and GFAP were observed in CA1 without an increase in adjacent brain regions, as shown in Figure 6, A. Overall, early adulthood CSS resulted in late-in-life evidence of glial activation within the HC, particularly in CA1. Having identified a reduction in CA1 volume without a parallel reduction in CA3 in response to CSS, we next compared the CSS Aβ and glial responses for CA1 and CA3. There were overall differences in glial responses for sleep conditions and HC region (DFd = 68, *F* = 6.5, *p* = 0.0004). Relative to Rest responses, increases were observed for Aβ (*df*=68, *t* = 6.5, *p* < 0.0001) and CD68 (*df* = 68, *t* = 3.2, *p* < 0.008) in CA1, relative to CA3, while Iba-1 and GFAP responses only trended towards increases in CA1 (as summarized in Figure 6, C). Overall, there were sleep and regional differences in the percentage of dystrophic microglia (DFd = 36, *F* = 16, *p* = 0.0003). Microglial morphology was distinct in CSS-exposed mice, with many more microglial cells in both CA1 showing fragmented projections, enlarged cell size, and/or spheroid tips on projections (*df* = 36, *q* = 12, *p* < 0.0001) and CA3 (*df* = 36, *q* = 4, *p* = 0.034); however, a greater percentage of dystrophic microglia was observed in CA1 than in CA3 (*df* = 36, *q* = 8.4, *p* < 0.0001) as summarized in Figure 6, D. Examples of dystrophic microglia are shown in Figure 6, E.

## Discussion

Extended periods of wake across school nights or during work weeks are common in modern society, where it is presumed that catch up sleep on weekends alleviates impairments following sleep loss. Experiments presented here demonstrate that chronic wake extension in the young adult mouse imparts significant late-in-life neural injury in parallel with a lasting hippocampal learning/memory impairment. Notably, the observed neural injury from CSS does not require any known genetic predisposition to a neurodegenerative process. The composite findings of CSS-induced loss of HC CA1 volume, loss of LC and CA1 neurons, glial activation, increased lateral ventricle volume and learning/memory impairment provide strong evidence that CSS can result in a neurodegenerative process, involving at least the LC and HC. While CSS in the WT mouse does not result in classic AD pathology, for example extracellular amyloid plaques and intraneuronal tau tangles, CSS results in strikingly long-term disturbances in amyloid and tau processing and abnormal glial activation, indicative of dramatic reprogramming of metabolic and inflammatory responses following young adult CSS exposure in addition to the irreversible injury.

LCn are susceptible to degeneration in AD [53, 54], but whether LCn loss is an important modulator in AD has not been established. Here, we found that 1 year after CSS exposure, LCn counts were reduced, relative to age-matched rested control mice. CSS of just 1 week duration also results in a loss of LCn of similar magnitude to the loss observed in the present study [33]. Collectively, these findings show that while there is no further loss of LCn during longer exposures to, or after, CSS, there is a clear persistence of loss, consistent with an irreversible degenerative process. LCn are the sole source of noradrenaline for the cortices [55], including the HC, and noradrenaline has been shown to suppress glial inflammatory responses [56, 57] and promote Aβ clearance [58]. Thus, the question arises, is a partial loss of LCn, in response to CSS, sufficient to explain the HC injury observed in the present study? In support, we find that reductions in LCn counts predicted reductions in CA1 counts, and in a previous study, LCn lesioning in a murine model of AD promoted glial activation, CA1 neuron loss and plaque density [59]. However, LCn lesioning in WT mice, did not modify glial response and CA1 neuron loss was not evident [59, 60]. Moreover, in WT rats, lesioning LCn reduced, rather than increased, Aβ _42_ in the forebrain, at least acutely [61]. Thus, we propose that while LCn loss in CSS may contribute to the observed neural injury, the composite CSS injury observed in the present study is more likely a multi-hit injury where additional CSS responses including increased Aβ _42_, tau phosphorylation and/or glial dysfunction may act in concert with LCn loss to promote late-in-life injury.

In young adult WT mice, acute sleep loss (across several hours) has been shown to increase extracellular Aβ _42_ in the HC, yet levels normalize upon recovery sleep [22]. In the present study, CSS resulted in increased Aβ in the HC, observed 1 year after CSS exposure, with a larger increase in CA1 relative to CA3, demonstrating both lasting disruptions in Aβ homeostasis and regional variations in the CSS Aβ response. The observed increase in HC Aβ in CSS mice 1 year after exposure indicates not only a persistent mismatch between Aβ production and clearance but additionally persistently inadequate clearance. The latter provides a clue to the mechanisms of the chronic disturbance. In the healthy brain, microglial cells play essential roles in Aβ clearance: uptake, degradation, and brain efflux [58], where soluble Aβ is taken into microglial cells by micropinocytosis and fibrillar Aβ by phagocytosis [62]. In the present study in CSS mice, Aβ was higher in CA1 than CA3. Dystrophy is used as an index of microglial dysfunction [63], and here we found that a greater percentage of microglia in CA1, relative to CA3, were dystrophic (fragmented, spheroid, and de-ramified processes). Microglial dysfunction is implicated in late-onset AD. Indeed, many of the genetic variants that strongly associate with AD are preferentially expressed in microglia [64–68]. We propose that CSS-induced microglial dysfunction and impaired uptake and clearance of Aβ may contribute to the lasting Aβ dyshomeostasis where circadian cycles of increased Aβ production across periods of spontaneous wakefulness may each be insufficiently cleared resulting in a gradual accumulation. Specifically, CSS may influence AD temporal progression and outcomes, at least in part, by disrupting microglial function.

Intriguingly, the CA1 region of the HC, is also affected acutely by sleep loss, where reductions in synaptic spine densities are appreciable after just 5 h of sleep deprivation, while CA3 appears largely unaffected [69]. Synapse loss in CA1 in response to acute sleep loss is, however, a reversible process in that after 3 h of recovery, CA1 sleep spine density normalizes [69]. Mechanisms underlying the acute CA1 synapse loss upon short-term sleep loss require activation of cofilin, a protein that breaks down actin infrastructure in synaptic spines [69]. Notably, Aβ oligomers also activate cofilin to reduce HC spine densities, and like acute sleep loss effects, spine density normalizes when Aβ is effectively cleared from the extracellular space [70]. Cofilin injury in AD, however, extends beyond synapse loss to include axonal transport deficits and mitochondrial injury, which may contribute to irreversible neural injury [71]. Here, we found that exposure to CSS in the young adult mouse resulted in late-in-life degeneration of CA1 neurons, as evidenced by both reduced cell counts and volume, and supported by regional gliosis and spatial memory impairment and that CA3 volume was less affected by CSS. While the goal of the present study was to define the extent of late-in-life HC injury after young adult CSS exposure, the observed CA1 differential vulnerability and commonality of Aβ in the context of established mechanisms of acute sleep loss CA1 injury unveil a likely pathway of injury to now explore.

It is not surprising that CSS mice developed neither amyloid plaques nor tau tangles. Species differences in Aβ sequence, and more importantly in APP processing of Aβ fragments, render the murine Aβ less prone to fibrillization and β-sheet formation, critical steps in the development of amyloid plaques [72]. Similarly, mice have alternate splicing differences in the tau isoforms, relative to humans, where normal WT adult mice lack tau 3R isoforms and do not develop intraneuronal aggregated tau. Nonetheless, work in the present study demonstrates that significant neural injury may occur in response to CSS, independent of amyloid plaques and tangles.

In conclusion, neural injury from exposure to CSS is evident a remarkably long time after exposure to sleep loss in the young adult, and the injury pattern shares some overlap with features in AD, yet occurs independently of the classic AD pathologies, tau tangles and amyloid plaques, lending further support to the concept that such pathologies are not necessary for significant neural injury. While the present findings in mice cannot be extrapolated to humans because of species differences in sleep patterns, Aβ and tau processing, and aging responses, the findings support early life sleep loss as a significant threat for late-in-life neural health and highlight the need to understand the long-term risks of chronic sleep curtailment in humans.

## References

[1] Scheltens P , et al Alzheimer’s disease. Lancet. 2016;388(10043):505–517. 26921134 10.1016/S0140-6736(15)01124-1

[2] Prince M , et al The global prevalence of dementia: a systematic review and metaanalysis. Alzheimers Dement. 2013;9(1):63–75.e2. 23305823 10.1016/j.jalz.2012.11.007

[3] Lane CA , et al Alzheimer’s disease. Eur J Neurol. 2018;25(1):59–70. 28872215 10.1111/ene.13439

[4] Spira AP , et al Self-reported sleep and β-amyloid deposition in community-dwelling older adults. JAMA Neurol. 2013;70(12):1537–1543. 24145859 10.1001/jamaneurol.2013.4258 PMC3918480

[5] Lucey BP , et al How amyloid, sleep and memory connect. Nat Neurosci. 2015;18(7):933–934. 26108720 10.1038/nn.4048 PMC4770804

[6] Bokenberger K , et al Association between sleep characteristics and incident dementia accounting for baseline cognitive status: a prospective population-based study. J Gerontol A Biol Sci Med Sci. 2017;72(1):134–139. 27402049 10.1093/gerona/glw127 PMC5155660

[7] Lim AS , et al Sleep fragmentation and the risk of incident Alzheimer’s disease and cognitive decline in older persons. Sleep. 2013;36 (7):1027–1032. 23814339 10.5665/sleep.2802 PMC3669060

[8] Itani O , et al Short sleep duration, shift work, and actual days taken off work are predictive life-style risk factors for new-onset metabolic syndrome: a seven-year cohort study of 40,000 male workers. Sleep Med. 2017;39:87–94. 29157594 10.1016/j.sleep.2017.07.027

[9] Bin YS , et al Sleeping at the limits: the changing prevalence of short and long sleep durations in 10 countries. Am J Epidemiol. 2013;177(8):826–833. 23524039 10.1093/aje/kws308

[10] Luckhaupt SE , et al The prevalence of short sleep duration by industry and occupation in the National Health Interview Survey. Sleep. 2010;33(2):149–159. 20175398 10.1093/sleep/33.2.149 PMC2817902

[11] Hardy JA , et al Alzheimer’s disease: the amyloid cascade hypothesis. Science. 1992;256(5054):184–185. 1566067 10.1126/science.1566067

[12] Hardy J . Testing times for the “amyloid cascade hypothesis”. Neurobiol Aging. 2002;23(6):1073–1074. 12470803 10.1016/s0197-4580(02)00042-8

[13] Hyman BT . Amyloid-dependent and amyloid-independent stages of Alzheimer disease. Arch Neurol. 2011;68(8):1062–1064. 21482918 10.1001/archneurol.2011.70

[14] Li S , et al Soluble oligomers of amyloid Beta protein facilitate hippocampal long-term depression by disrupting neuronal glutamate uptake. Neuron. 2009;62(6):788–801. 19555648 10.1016/j.neuron.2009.05.012 PMC2702854

[15] Kopeikina KJ , et al Soluble forms of tau are toxic in Alzheimer’s disease. Transl Neurosci. 2012;3(3):223–233. 23029602 10.2478/s13380-012-0032-y PMC3460520

[16] Mucke L , et al Neurotoxicity of amyloid β-protein: synaptic and network dysfunction. Cold Spring Harb Perspect Med. 2012;2(7):a006338. 22762015 10.1101/cshperspect.a006338 PMC3385944

[17] Menkes-Caspi N , et al Pathological tau disrupts ongoing network activity. Neuron. 2015;85(5):959–966. 25704951 10.1016/j.neuron.2015.01.025

[18] Spires-Jones TL , et al Interactions of pathological proteins in neurodegenerative diseases. Acta Neuropathol. 2017;134(2):187–205. 28401333 10.1007/s00401-017-1709-7 PMC5508034

[19] Roberson ED , et al Reducing endogenous tau ameliorates amyloid beta-induced deficits in an Alzheimer’s disease mouse model. Science. 2007;316(5825):750–754. 17478722 10.1126/science.1141736

[20] Ittner LM , et al Dendritic function of tau mediates amyloid-beta toxicity in Alzheimer’s disease mouse models. Cell. 2010;142(3):387–397. 20655099 10.1016/j.cell.2010.06.036

[21] DeVos SL , et al Tau reduction in the presence of amyloid-β prevents tau pathology and neuronal death in vivo. Brain. 2018;141(7):2194–2212. 29733334 10.1093/brain/awy117 PMC6022692

[22] Kang JE , et al Amyloid-beta dynamics are regulated by orexin and the sleep-wake cycle. Science. 2009;326(5955):1005–1007. 19779148 10.1126/science.1180962 PMC2789838

[23] Roh JH , et al Disruption of the sleep-wake cycle and diurnal fluctuation of β-amyloid in mice with Alzheimer’s disease pathology. Sci Transl Med. 2012;4(150):150ra122. 10.1126/scitranslmed.3004291 PMC3654377 22956200

[24] Holth JK , et al The sleep-wake cycle regulates brain interstitial fluid tau in mice and CSF tau in humans. Science. 2019;363(6429):880–884. 30679382 10.1126/science.aav2546 PMC6410369

[25] Li X , et al Neuronal activity and secreted amyloid β lead to altered amyloid β precursor protein and presenilin 1 interactions. Neurobiol Dis. 2013;50:127–134. 23064434 10.1016/j.nbd.2012.10.002 PMC3534898

[26] Leal SL , et al Hippocampal activation is associated with longitudinal amyloid accumulation and cognitive decline. eLife. 2017;6:e22978. 28177283 10.7554/eLife.22978 PMC5325620

[27] Xie L , et al Sleep drives metabolite clearance from the adult brain. Science. 2013;342(6156):373–377. 24136970 10.1126/science.1241224 PMC3880190

[28] Zott B , et al A vicious cycle of β amyloid-dependent neuronal hyperactivation. Science. 2019;365(6453):559–565. 31395777 10.1126/science.aay0198 PMC6690382

[29] Opazo P , et al CaMKII Metaplasticity drives Aβ Oligomer-mediated Synaptotoxicity. Cell Rep. 2018;23(11):3137–3145. 29898386 10.1016/j.celrep.2018.05.036 PMC6089247

[30] McCullough LD , et al Ischemic nitric oxide and poly (ADP-ribose) polymerase-1 in cerebral ischemia: male toxicity, female protection. J Cereb Blood Flow Metab. 2005;25(4):502–512. 15689952 10.1038/sj.jcbfm.9600059

[31] Gompf HS , et al Locus ceruleus and anterior cingulate cortex sustain wakefulness in a novel environment. J Neurosci. 2010;30(43):14543–14551. 20980612 10.1523/JNEUROSCI.3037-10.2010 PMC2989851

[32] Léger L , et al Noradrenergic neurons expressing Fos during waking and paradoxical sleep deprivation in the rat. J Chem Neuroanat. 2009;37(3):149–157. 19152834 10.1016/j.jchemneu.2008.12.008

[33] Zhang J , et al Extended wakefulness: compromised metabolics in and degeneration of locus ceruleus neurons. J Neurosci. 2014;34(12):4418–4431. 24647961 10.1523/JNEUROSCI.5025-12.2014 PMC3960479

[34] Zhu Y , et al Intermittent short sleep results in lasting sleep wake disturbances and degeneration of locus coeruleus and orexinergic Neurons. Sleep. 2016;39(8):1601–1611. 27306266 10.5665/sleep.6030 PMC4945320

[35] Panossian L , et al SIRT1 regulation of wakefulness and senescence-like phenotype in wake neurons. J Neurosci. 2011;31(11):4025–4036. 21411645 10.1523/JNEUROSCI.5166-10.2011 PMC3065120

[36] Bachstetter AD , et al Disease-related microglia heterogeneity in the hippocampus of Alzheimer’s disease, dementia with Lewy bodies, and hippocampal sclerosis of aging. Acta Neuropathol Commun. 2015;3:32. 26001591 10.1186/s40478-015-0209-z PMC4489160

[37] West MJ , et al Unbiased stereological estimation of the number of neurons in the human hippocampus. J Comp Neurol. 1990;296(1):1–22. 2358525 10.1002/cne.902960102

[38] Schaeffer EL , et al Stereological investigation of the CA1 pyramidal cell layer in untreated and lithium-treated 3xTg-AD and wild-type mice. Ann Anat. 2017;209:51–60. 27777112 10.1016/j.aanat.2016.10.002

[39] Vogel-Ciernia A , et al Examining object location and object recognition memory in mice. Curr Protoc Neurosci. 2014;69:8.31.1–8.3117. 10.1002/0471142301.ns0831s69 PMC4219523 25297693

[40] Braak H , et al Neuropathological stageing of Alzheimer-related changes. Acta Neuropathol. 1991;82(4):239–259. 1759558 10.1007/BF00308809

[41] West MJ , et al Differences in the pattern of hippocampal neuronal loss in normal ageing and Alzheimer’s disease. Lancet. 1994;344(8925):769–772. 7916070 10.1016/s0140-6736(94)92338-8

[42] Kelly SC , et al Locus coeruleus cellular and molecular pathology during the progression of Alzheimer’s disease. Acta Neuropathol Commun. 2017;5(1):8. 28109312 10.1186/s40478-017-0411-2 PMC5251221

[43] Morris RG , et al Place navigation impaired in rats with hippocampal lesions. Nature. 1982;297(5868):681–683. 7088155 10.1038/297681a0

[44] McGregor A , et al Hippocampal lesions disrupt navigation based on the shape of the environment. Behav Neurosci. 2004;118(5):1011–1021. 15506883 10.1037/0735-7044.118.5.1011

[45] Apostolova LG , et al 3D comparison of hippocampal atrophy in amnestic mild cognitive impairment and Alzheimer’s disease. Brain. 2006;129(Pt 11):2867–2873. 17018552 10.1093/brain/awl274

[46] Hong S , et al Complement and microglia mediate early synapse loss in Alzheimer mouse models. Science. 2016;352(6286):712–716. 27033548 10.1126/science.aad8373 PMC5094372

[47] Zhu Y , et al Chronic sleep disruption advances the temporal progression of tauopathy in P301S mutant mice. J Neurosci. 2018;38(48):10255–10270. 30322903 10.1523/JNEUROSCI.0275-18.2018 PMC6262148

[48] Stranahan AM , et al Selective vulnerability of neurons in layer II of the entorhinal cortex during aging and Alzheimer’s disease. Neural Plast. 2010;2010:108190. 21331296 10.1155/2010/108190 PMC3039218

[49] Adams JN , et al Cortical tau deposition follows patterns of entorhinal functional connectivity in aging. eLife. 2019;8:e49132. 31475904 10.7554/eLife.49132 PMC6764824

[50] Braak H , et al Stages of the pathologic process in Alzheimer disease: age categories from 1 to 100 years. J Neuropathol Exp Neurol. 2011;70(11):960–969. 22002422 10.1097/NEN.0b013e318232a379

[51] Buerger K , et al Differential diagnosis of Alzheimer disease with cerebrospinal fluid levels of tau protein phosphorylated at threonine 231. Arch Neurol. 2002;59(8):1267–1272. 12164722 10.1001/archneur.59.8.1267

[52] Kondo A , et al Antibody against early driver of neurodegeneration cis P-tau blocks brain injury and tauopathy. Nature. 2015;523(7561):431–436. 26176913 10.1038/nature14658 PMC4718588

[53] Tomlinson BE , et al Cell loss in the locus coeruleus in senile dementia of Alzheimer type. J Neurol Sci. 1981;49(3):419–428. 7217992 10.1016/0022-510x(81)90031-9

[54] Bondareff W , et al Loss of neurons of origin of the adrenergic projection to cerebral cortex (nucleus locus ceruleus) in senile dementia. Neurology. 1982;32(2):164–168. 7198741 10.1212/wnl.32.2.164

[55] Aston-Jones G , et al Activity of norepinephrine-containing locus coeruleus neurons in behaving rats anticipates fluctuations in the sleep-waking cycle. J Neurosci. 1981;1(8):876–886. 7346592 10.1523/JNEUROSCI.01-08-00876.1981 PMC6564235

[56] Hu XX , et al The effect of norepinephrine on endotoxin-mediated macrophage activation. J Neuroimmunol. 1991;31(1):35–42. 1845768 10.1016/0165-5728(91)90084-k

[57] Feinstein DL , et al Noradrenergic regulation of inflammatory gene expression in brain. Neurochem Int. 2002;41(5):357–365. 12176079 10.1016/s0197-0186(02)00049-9

[58] Bolmont T , et al Dynamics of the microglial/amyloid interaction indicate a role in plaque maintenance. J Neurosci. 2008;28(16):4283–4292. 18417708 10.1523/JNEUROSCI.4814-07.2008 PMC3844768

[59] Heneka MT , et al Locus ceruleus degeneration promotes Alzheimer pathogenesis in amyloid precursor protein 23 transgenic mice. J Neurosci. 2006;26(5):1343–1354. 16452658 10.1523/JNEUROSCI.4236-05.2006 PMC6675491

[60] Chalermpalanupap T , et al Locus coeruleus ablation exacerbates cognitive deficits, neuropathology, and lethality in P301S tau transgenic mice. J Neurosci. 2018;38(1):74–92. 29133432 10.1523/JNEUROSCI.1483-17.2017 PMC5761438

[61] Ross JA , et al Localization of endogenous amyloid-β to the coeruleo-cortical pathway: consequences of noradrenergic depletion. Brain Struct Funct. 2018;223(1):267–284. 28779307 10.1007/s00429-017-1489-9 PMC5773352

[62] Mandrekar S , et al Microglia mediate the clearance of soluble Abeta through fluid phase macropinocytosis. J Neurosci. 2009;29(13):4252–4262. 19339619 10.1523/JNEUROSCI.5572-08.2009 PMC3034143

[63] Mosher KI , et al Microglial dysfunction in brain aging and Alzheimer’s disease. Biochem Pharmacol. 2014;88(4):594–604. 24445162 10.1016/j.bcp.2014.01.008 PMC3972294

[64] Strittmatter WJ , et al Apolipoprotein E: high-avidity binding to beta-amyloid and increased frequency of type 4 allele in late-onset familial Alzheimer disease. Proc Natl Acad Sci USA. 1993;90(5):1977–1981. 8446617 10.1073/pnas.90.5.1977 PMC46003

[65] Pottier C , et al; PHRC GMAJ Collaborators. High frequency of potentially pathogenic SORL1 mutations in autosomal dominant early-onset Alzheimer disease. Mol Psychiatry. 2012;17(9):875–879. 22472873 10.1038/mp.2012.15

[66] Guerreiro R , et al; Alzheimer Genetic Analysis Group. TREM2 variants in Alzheimer’s disease. N Engl J Med. 2013;368(2):117–127. 23150934 10.1056/NEJMoa1211851 PMC3631573

[67] Jonsson T , et al Variant of TREM2 associated with the risk of Alzheimer’s disease. N Engl J Med. 2013;368(2):107–116. 23150908 10.1056/NEJMoa1211103 PMC3677583

[68] Steinberg S , et al; DemGene. Loss-of-function variants in ABCA7 confer risk of Alzheimer’s disease. Nat Genet. 2015;47(5):445–447. 25807283 10.1038/ng.3246

[69] Havekes R , et al Sleep deprivation causes memory deficits by negatively impacting neuronal connectivity in hippocampal area CA1. eLife. 2016;5:e13424. 27549340 10.7554/eLife.13424 PMC4996653

[70] Shankar GM , et al Natural oligomers of the Alzheimer amyloid-beta protein induce reversible synapse loss by modulating an NMDA-type glutamate receptor-dependent signaling pathway. J Neurosci. 2007;27(11):2866–2875. 17360908 10.1523/JNEUROSCI.4970-06.2007 PMC6672572

[71] Maloney MT , et al Cofilin-mediated neurodegeneration in Alzheimer’s disease and other amyloidopathies. Mol Neurobiol. 2007;35(1):21–44. 17519504 10.1007/BF02700622

[72] Xu G , et al Murine Aβ over-production produces diffuse and compact Alzheimer-type amyloid deposits. Acta Neuropathol Commun. 2015;3:72. 26566997 10.1186/s40478-015-0252-9 PMC4644287

